# Molecular evidence for ongoing complementarity and horizontal gene transfer in endosymbiotic systems of mealybugs

**DOI:** 10.3389/fmicb.2014.00449

**Published:** 2014-08-26

**Authors:** Sergio López-Madrigal, Aleixandre Beltrà, Serena Resurrección, Antonia Soto, Amparo Latorre, Andrés Moya, Rosario Gil

**Affiliations:** ^1^Institut Cavanilles de Biodiversitat i Biologia Evolutiva, Universitat de ValenciaValencia, Spain; ^2^Instituto Agroforestal Mediterráneo, Universitat Politecnica de ValenciaValencia, Spain; ^3^Área de Genómica y Salud de la Fundación para el Fomento de la Investigación Sanitaria y Biomédica de la Comunitat Valenciana (FISABIO)–Salud PúblicaValencia, Spain

**Keywords:** mealybugs, endosymbiosis, “*Candidatus* Tremblaya princeps”, “*Candidatus* Tremblaya phenacola”, amino acid biosynthesis, horizontal gene transfer

## Abstract

Intracellular bacterial supply of essential amino acids is common among sap-feeding insects, thus complementing the scarcity of nitrogenous compounds in plant phloem. This is also the role of the two mealybug endosymbiotic systems whose genomes have been sequenced. In the nested endosymbiotic system from *Planococcus citri* (Pseudococcinae), “*Candidatus* Tremblaya princeps” and “*Candidatus* Moranella endobia” cooperate to synthesize essential amino acids, while in *Phenacoccus avenae* (Phenacoccinae) this function is performed by its single endosymbiont “*Candidatus* Tremblaya phenacola.” However, little is known regarding the evolution of essential amino acid supplementation strategies in other mealybug systems. To address this knowledge gap, we screened for the presence of six selected loci involved in essential amino acid biosynthesis in five additional mealybug species. We found evidence of ongoing complementarity among endosymbionts from insects of subfamily Pseudococcinae, as well as horizontal gene transfer affecting endosymbionts from insects of family Phenacoccinae, providing a more comprehensive picture of the evolutionary history of these endosymbiotic systems. Additionally, we report two diagnostic motifs to help identify invasive mealybug species.

## Introduction

The establishment of permanent intracellular symbioses with bacteria has played a key role in insect evolution. Endosymbionts are located in specialized eukaryote cells (bacteriocytes) and complement the insect's limited heterotrophic metabolism with metabolic pathways for the biosynthesis of essential amino acids, fatty acids and/or vitamins (Baumann, 2005). Population dynamics imposed by their lifestyle, together with the stability and nutritional richness of the intracellular environment, trigger a number of genomic changes in the prokaryote symbiont. Such evolutionary changes include drastic genome size reduction, due to the loss of genes rendered unnecessary for the association (i.e., those that are superfluous in a protected and stable intracellular niche or whose function can be provided by the host), and an increase in AT content in most analyzed cases (Baumann, 2005; Moran et al., 2008; Moya et al., 2008; Gil et al., 2010). Eventually, if a second bacterium joins the association, both bacteria coevolve and the ongoing reductive genome process affects both of them, leading to two possible outcomes: either both bacteria become essential for the fitness of the association (complementation), or one bacterium undergoes an extreme genome degenerative process, which may end up in its extinction, while the remaining bacterium continues the reductive process alone (replacement) (Moya et al., 2009).

As in other phloem-feeding insects, mealybugs rely on their endosymbionts for the provision of essential amino acids (Baumann, 2005). This fact is supported by the recent genome sequencing of endosymbionts from two mealybug species belonging to subfamilies Pseudococcinae and Phenacoccinae (Lopez-Madrigal et al., 2011; McCutcheon and von Dohlen, 2011; Husnik et al., 2013). Mealybugs from these subfamilies present an intricate variety of endosymbiotic relationships that reflect both complementation and replacement events. Phylogenetic studies suggest that a betaproteobacterial ancestor of “*Ca*. Tremblaya” infected a mealybug ancestor before the split of subfamilies Phenacoccinae and Pseudococcinae (Hardy et al., 2008). Later on, except for the *Ferrisia* and *Maconellicoccus* clades (where no additional endosymbiont has been reported), the ancestor of “*Ca*. Tremblaya princeps” was infected multiple times by different gammaproteobacteria, establishing a diversity of stable nested endosymbiotic consortia, with each “*Ca*. Tremblaya princeps” cell containing several cells of the corresponding gammaproteobacterium (Thao et al., 2002; Gatehouse et al., 2012). By contrast, “*Ca*. Tremblaya phenacola” remained alone in subfamily Phenacoccinae, except in several clades (the tribe *Rhizoecini* and the genus *Rastrococcus*) where it was replaced by different *Bacteroidetes* (Gruwell et al., 2010; Husnik et al., 2013).

The most widely studied mealybug endosymbiotic system belongs to *Planococcus citri* (Risso), where “*Ca*. Tremblaya princeps” harbors “*Ca*. Moranella endobia” (McCutcheon and von Dohlen, 2011), with a tight relationship between the nested endosymbiosis dynamics and the insect life-cycle (von Dohlen et al., 2001; Kono et al., 2008). Independent genomic studies on the endosymbiotic consortium of two *P. citri* strains (PCIT and PCVAL) revealed their entangled metabolic complementation for the biosynthesis of some essential amino acids (McCutcheon and von Dohlen, 2011; Lopez-Madrigal et al., 2013), and necessary participation of the insect host (Husnik et al., 2013). Husnik and coworkers also reported horizontal gene transfer (HGT) of some genes involved in these pathways from diverse bacteria to the insect nuclear genome. Even though “*Ca*. Tremblaya princeps” from *P. citri* exhibits one of the smallest prokaryote genomes known so far (139 kb), with an extremely reduced gene set, it is the only source for at least 29 enzymes needed to synthesize several essential amino acids (Lopez-Madrigal et al., 2011; McCutcheon and von Dohlen, 2011). Functional redundancy in both endosymbiotic partners was observed only for *dapA* (involved in lysine biosynthesis) and *aroK* (involved in phenylalanine and tryptophan biosynthesis), an indication that each bacterium has adopted a specific role in essential amino acid provision. Recent sequencing of “*Ca*. Tremblaya phenacola” PAVE, the sole endosymbiont of *Phenacoccus avenae* (Borchsenius), revealed a remarkable case of evolutionary convergence, since it has preserved exactly the same set of genes, collectively retained by “*Ca*. Tremblaya princeps” and “*Ca*. Moranella endobia” in *P. citri*, for supplying essential amino acids to the host (Husnik et al., 2013).

In order to study the evolution of essential amino acids provisioning within unexplored “*Ca*. Tremblaya” lineages, we have performed a genetic screening on several mealybug species, including members of both subfamily Pseudococcinae (*Dysmicoccus boninsis* Kuwana, *Pseudococcus viburni* Signoret and *Pseudococcus longispinus* Targioni-Tozzetti) and subfamily Phenacoccinae (*Phenacoccus peruvianus* Granara de Willink and *Phenacoccus madeirensis* Green). We searched for the presence of genes *argH*, *ilvD*, *leuB*, *metE*, *thrC*, and *trpB*, encoding the enzymes that participate in the last steps performed by the *P. citri* and *P. avenae* endosymbiotic systems in the pathways for the biosynthesis of arginine, branched amino acids, methionine, threonine and tryptophan, respectively. We have also characterized the endosymbionts present in these mealybug species at the molecular and phylogenetic level. Our results reveal differences among different clades, both at the molecular and functional levels, thus providing a more complete picture on the complex evolutionary history of the two “*Ca*. Tremblaya” lineages.

## Materials and methods

### Insect sample collection and DNA extraction

Insects belonging to the species *D. boninsis*, *P. viburni*, *P. longispinus*, *P. peruvianus*, and *P. madeirensis* were field collected in Valencia (Spain) and stored in absolute ethanol at −20°C. Total DNA (_T_DNA) extractions were performed with JETFLEX Genomic DNA Purification Kit (GENOMED) on 5–8 adult females.

### DNA amplification, sequencing and analysis

PCR amplifications were performed on insect _T_DNA, with appropriate primer pairs (see Section Gene Screening), using 50–60 μmol of each primer/50 μl reaction, with KAPATaq DNA Polymerase Kit (Kapa Biosystems). The thermal cycling protocol was as follows: an initial denaturation at 95°C for 5 min, followed by 35 cycles of 50 s at 95°C, 40 s at 56°C (or 52°C when indicated), and 2 min at 72°C, plus a final extension step of 7 min at 72°C. When needed, amplicons were cloned using pGEM-T Easy Vector System I Kit (Promega). ABI sequencing was performed, using specific or vector primers T7 and SP6, at the sequencing facility of the Universitat de València. Sequencing reads were quality surveyed and assembled with Staden Package (http://staden.sourceforge.net/; Staden et al., 2000). Artemis software was used for sequence data management (http://www.sanger.ac.uk/resources/software/artemis/; Rutherford et al., 2000). Multiple alignments were performed with ClustalW (Larkin et al., 2007). MEGA5 was used for the calculation of both p-distance and nucleotide composition.

### Gene screening

Complete sequences of *argH* (encoding argininosuccinate lyase, EC 4.3.2.1, involved in arginine biosyntesis), *ilvD* (encoding dihydroxy-acid dehydratase, EC 4.2.1.9, involved in isoleucine and valine biosynthesis), *leuB* (encoding 3-isopropylmalate dehydrogenase, EC 1.1.1.85, involved in leucine biosynthesis), *metE* (encoding cobalamin-independent homocysteine transmethylase, EC 2.1.1.14, involved in methionine biosynthesis), *thrC* (encoding threonine synthase, EC 4.2.3.1, involved in threonine biosynthesis), and *trpB* (encoding the beta subunit of tryptophan synthase, EC 4.2.1.20, involved in tryptophan biosynthesis), were retrieved from GenBank for a set of selected beta and gammaproteobacteria (Table S1 in Supplementary Material). Multiple alignments were performed in ClustalW to allow the design of degenerate primers (Table 1) to amplify the corresponding gene by PCR, using both 52°C and 56°C as annealing temperature (T_a_) for all primer pairs. _T_DNA from *P. citri* was used as a positive control. At least 10 clones for each amplicon were sequenced. BLAST searches against the non-redundant protein database (http://blast.ncbi.nlm.nih.gov/Blast.cgi/; Altschul et al., 1997) were performed in order to identify the putative taxonomic origin of the sequences obtained.

**Table 1:** **Degenerate primers used in the gene screening**.

**Gene**	**Primer**	**Sequence (5′ →3′)**	Degeneracy^*^ (%)	**Conserved motif**
*argH*	argH-F	AAYGAYCARRTNGCNACNGA	35	NDQ(V/I)ATD
	argH-R	TCNGGRTTYTTYTTYTGNGGCAT	26	MPQKKNPD
*ilvD*	ilvD-F	ATGTWYACVGCNAAYWCNATG	33	M(F/Y)TAN(S/T)M
	ilvD-R	GARAARAANCKVCCRTCNGT	35	SGGT(S/W)G
*leuB*	leuB-F	GGNGAYGGHATHGGBCCBGA	30	GDGIGPE
	leuB-R	TCVGAVARDATRTCGCCRAA	30	FGDILSD
*metE*	metE-F	AACTAYCACTAYMTVGTVCCNGA	26	NYHY(M/I/L)VPE
	metE-R	CCRCARTCHGGRTTNAYCCA	30	W(V/I)NPDCG
*thrC*	thrC-F	GCDACNTCNGGBGAYACNGG	30	ATSGDTG
	thrC-R	TYNCCRAARTTNCCNSWNGG	45	PSGNFG(D/N)
*trpB*	trpB-F	GTNHTNGGNCARGCNYTNYTNGC	43	V(L/I)GQALLA
	trpB-R	TCNCCNCGNCCNGANAGRTT	30	NLSGRGD

* *Primer degeneracy was measured as the proportion of ambiguous sites*.

_T_DNA from *D. boninsis*, *P. longispinus*, *P. peruvianus*, and *P. madeirensis* were used as templates for PCR amplification using 16S rDNA universal primers (Table S2 in Supplementary Material; van Ham et al., 1997). The amplicons were cloned as above mentioned, and at least 25 clones were sequenced for each species. The newly found sequences have been deposited in the GenBank database (see Table S3 in Supplementary Material). Samples from *P. peruvianus* and *P. madeirensis* were also analyzed by PCR using gammaproteobacteria-specific 16S rDNA primers (Mühling et al., 2008).

In order to determine the location of the *trpB* gene in *P. peruvianus*, we dissected three adult females to separate the head from the rest of the body, and extracted _T_DNA from both samples. Universal primers were used to amplify 18S rDNA from the host genome (T_a_ = 52°C), as PCR positive control (Table S2 in Supplementary Material; Littlewood and Olson, 2001). We designed two sets of specific primers to amplify 16S rDNA from “*Ca*. Tremblaya phenacola” and the *trpB* sequence found in *P. peruvianus*, respectively. All PCR products were sequenced using the same primers to confirm their identity.

A diagnostic screening by restriction enzyme analysis was performed on the 16S rRNA genes amplified from *P. longispinus*, in order to check for the putative presence of other gammaproteobacterial haplotypes previously identified in this species (Duron et al., 2008; Rosenblueth et al., 2012). We designed a pair of primers in a conserved region of the 16S rDNA sequences of the three gammaproteobacterial haplotypes and “*Ca*. Tremblaya princeps” from *P. longispinus* (Table S2 in Supplementary Material). RFLP-up and RFLP-down amplify the region from sites 516 to 1075 in the *E. coli* K-12 *substr*. MG1655 homolog. PCR products were digested with the enzyme *Rsa*I (Roche). Restriction digest products were run on an agarose gel, stained in ethidium bromide, and visualized with UV light.

### Fluorescence *in situ* hybridization (fish)

Field collected *P. peruvianus* adult females were decapitated and placed in 4% paraformaldehyde for fixation. Samples were stored at 4°C in phosphate buffer saline (PBS) with 0.05% azide until preparation for paraffin inclusion. To do so, samples were dehydrated through a graded ethanol series, from 70 to 100% ethanol, and finally washed twice in xylene at room temperature for 30 min. Then, they were embedded in paraffin and cut on a microtome into 5 μm thick sections, placed on poly-lysine coated slides, air dried, and kept at 4°C. Prior to usage, paraffin sections were dewaxed in two xylene baths, followed by two absolute ethanol baths of 10 min each.

In order to permeabilize cellular membranes, samples were coated with a few drops of 70% acetic acid while incubated on a 60°C hotplate for 1 min. Once rinsed with PBS, they were dehydrated again through a graded ethanol series and air-dried. Slides were subsequently coated with 100 μl of hybridization buffer (Tris 20 mM pH 8, NaCl 0.9 M, SDS 0.01% and formamide 30%) plus 100 ng of the 16S rDNA universal probe Cy5-EUB338 (Amann et al., 1990) or the specific probe Cy3-TphPPER1290 (5′-CCGCAATTCGTACTGAGGTTAGG-3′), designed on “*Ca*. Tremblaya phenacola” PPER 16S rRNA gene, and incubated for 3 h at 45°C. To confirm hybridization signal specificity, the following control experiments were performed: a no-probe control, an RNase digestion control (slides treated for 30 min with RNase A prior to hybridization), and a competitive suppression control with excess unlabeled probe (80 ng/μl of hybridization buffer) (Fukatsu et al., 1998). In order to preserve fluorescent signal, slides were kept in dark from this point on. After hybridization, they were coated with 1 μg/mL DAPI for 10 min. Four 10-min washes were performed with washing buffer (Tris 20 mM pH 8, NaCl 112 mM, SDS 0.01% and EDTA 5 mM) at 48°C. Finally, samples were rinsed twice with milliQ water for 5 min at room temperature. Once completely air-dried, slides were mounted with Fluoromount-G (Southern Biotech) and kept at 4°C overnight.

Slides were observed under an epifluorescence microscope (Nikon Eclipse 80i). Nikon DS-Qi1Mc digital camera and NIS-Elements BR 3.0 software were used for image capturing and processing, respectively.

### Phylogenetic analyses

Nucleotide sequences used for the phylogenetic analysis were retrieved from GenBank or obtained in this work. The complete list of sequences, from selected alpha, beta and gammaproteobacteria (including both endosymbiotic and free-living species) is presented in Table S3 in Supplementary Material.

Phylogenetic reconstructions were carried out by Maximun Likelihood (ML), Maximum Parsimony (MP) and Bayesian methods, in RAxML (Stamatakis, 2006), DNAPARS from PHYLIP v3.69 package (Felsenstein, 2005), and MrBayes 3.2 (Ronquist et al., 2012), respectively. According to JModelTest (Guindon and Gascuel, 2003; Darriba et al., 2012), we applied a separate general time-reversible evolutionary model with gamma-distributed rates and a proportion of invariant sites (GTR+I+G) in phylogenetic reconstructions by ML and Bayesian methods. In ML and MP reconstructions, bootstrap analyses were performed with 1000 replications. In Bayesian reconstructions, phylogenetic trees were generated from two runs of 200,000 generations for 16S rDNA and two runs of 500,000 generations for *trpB* and *trpB-argH*. Likelihood settings were set to nst = 6, rates = invgamma and ngammacat = 4. Sampling was performed every 100 generations. First 2300, 3200, and 3500 generations were discarded as “burn in” for runs on *trpB*-*argH*, 16S rDNA and *trpB* molecular data, respectively. Figures on phylogenetic analysis were prepared with FigTree v1.4.0 software (http://tree.bio.ed.ac.uk/software/figtree/).

## Results

### Screening of genes involved in essential amino acids biosynthesis

**Figure 1:**
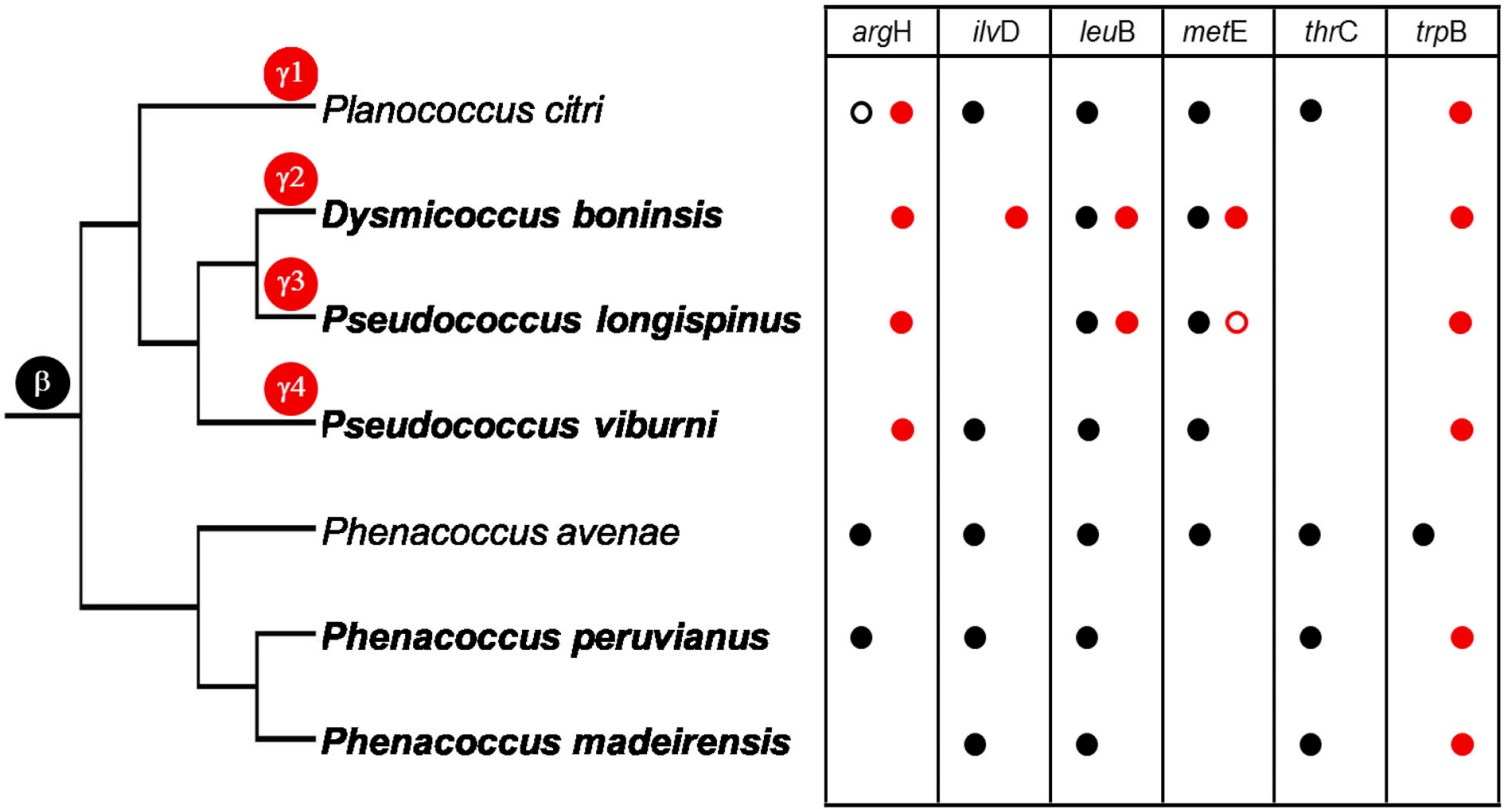
**Genetic screening of selected loci involved in essential amino acid biosynthesis**. The taxonomic assignation of the amplified sequences is indicated by black (betaproteobacteria) and red (gammaproteobacteria) circles. Empty circles represent pseudogenes. The species analyzed in this work appear in bold. *P. citri* and *P. avenae* endosymbionts are shown for comparison. Cladogram topology represents the evolutionary relationships between insect lineages (based on Hardy et al., 2008; Gruwell et al., 2010; this work). Marks on the branches indicate the infection by the ancestor of “*Ca*. Tremblaya” (β) and different lineages of gammaproteobacteria. γ1 corresponds to “*Ca*. Moranella endobia.” γ2–4 correspond to non-monophyletic gammaproteobacteria, based in our phylogenetic analyses.

The putative capability for essential amino acid biosynthesis among the analyzed species was evaluated by a PCR screening of selected genes involved in the last step usually performed by the mealybug endosymbiotic systems in the biosynthetic pathways of most essential amino acids. We did not attempt to detect genes involved in the biosynthesis of histidine and phenylalanine given the impracticality of designing reliable degenerate primers on genes *hisB* and *pheA*, due to their considerably smaller length and/or conservation level. The results of this genetic screening are shown in Figure 1.

*argH* of gammaproteobacterial origin, according to BLAST results, was found in all analyzed Pseudococcinae lineages. Although “*Ca*. Tremblaya princeps” from *P. citri* also contains an *argH* homolog, it is pseudogenized (Lopez-Madrigal et al., 2011; McCutcheon and von Dohlen, 2011). Locus degeneration is evidenced by two deletions, involving 6 and 57 nucleotides, affecting a highly conserved region in analyzed betaproteobacteria and *E. coli* (gammaproteobacterium), as well as by an inactivating frameshift caused by a single cytosine deletion (see Supplementary Material). An *argH* homolog of betaproteobacterial origin was also detected in *P. peruvianus*, but not in the *P. madeirensis* sample.

Regarding *ilvD*, *leuB* and *metE*, *P. viburni* resembles *P. citri*, since they both contain orthologs solely of betaproteobacterial origin. In contrast, only an *ilvD* gene of gammaproteobacterial origin was detected in *D. boninsis*, while no functional homolog was detected in *P. longispinus*. Loci *leuB* and *metE* were redundant both in *D. boninsis* and *P. longispinus*, although the gammaproteobacterial *metE* homolog in *P. longispinus* is apparently inactivated due to a non-sense mutation (TGG→TAG) affecting a highly conserved residue (W140 in the *E. coli* homolog protein). Nevertheless, all the other important residues for protein functioning examined are still preserved (Table S4 in Supplementary Material). MetE key residues identified in *E. coli* are preserved in *Burkholderia*, the closest free-living betaproteobacterial relative of “*Ca*. Tremblaya,” and they are also present in all homolog proteins of the different “*Ca*. Tremblaya princeps” strains analyzed. Only a non-synonymous Ile→Val change was observed in all of them. Nonetheless, this change is not expected to have functional consequences, given the similarities in size and polarity of both amino acids. However, this gene was not detected in the two Phenococcinae analyzed in this work.

In the case of *thrC*, we could not detect this gene in other “*Ca*. Tremblaya princeps” except that from *P. citri*, while it was identified in the two sampled “*Ca*. Tremblaya phenacola.” Finally, only a gammaproteobacterial homolog of *trpB* was found in all analyzed mealybugs.

**Figure 2:**
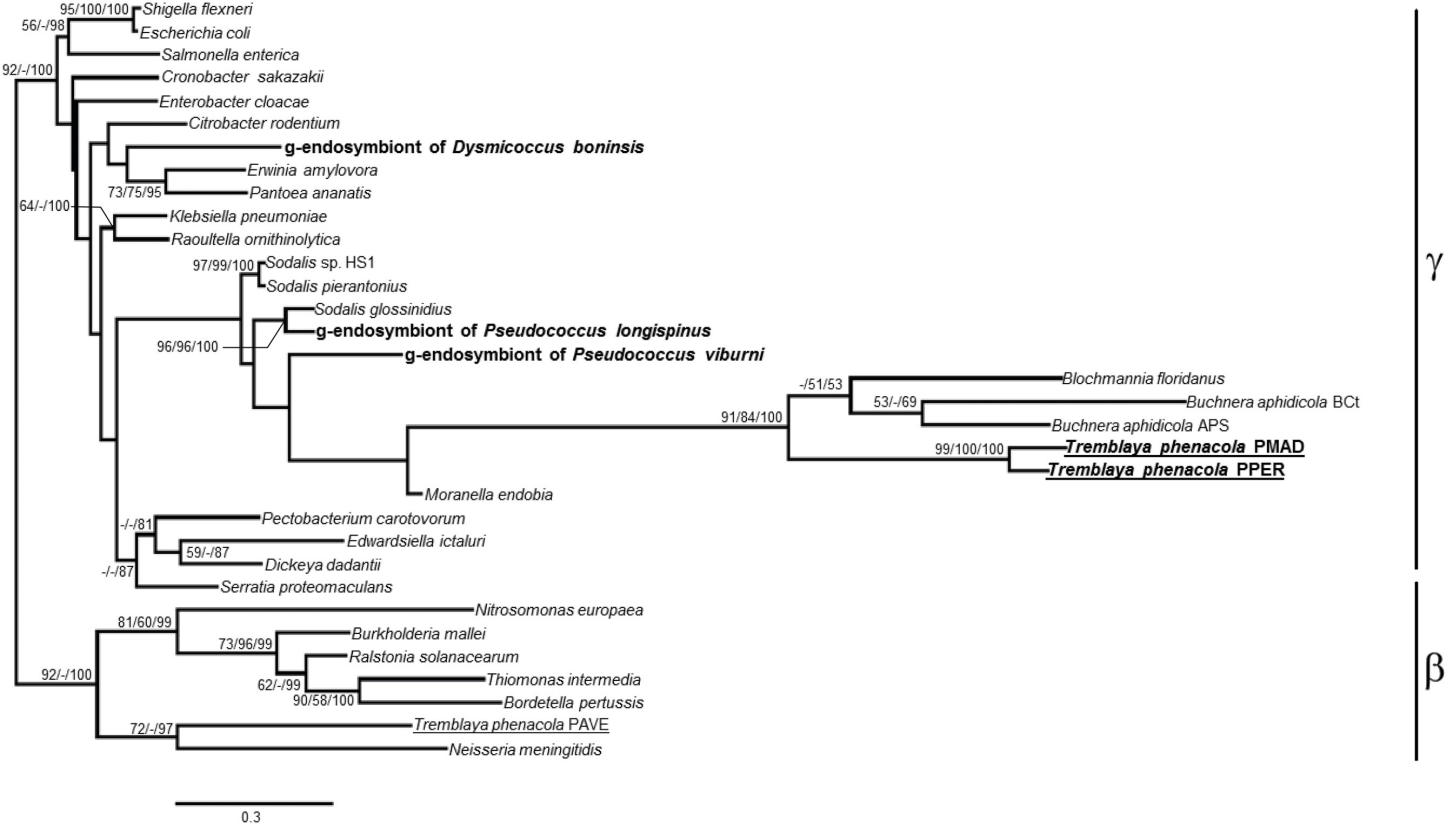
**ML phylogenetic analysis of the *trpB* partial nucleotide sequences obtained from *P. peruvianus* and *P. madeirensis* samples**. Sequences obtained in this work are in bold; those from the Phenacoccinae are underlined. Bayesian and MP analysis gave essentially the same results. ML and MP bootstrap values, and Bayesian posterior probabilities over 50% are represented. Scale bar represents substitutions per site.

As expected for mealybugs of subfamily Phenacoccinae, most of the amplified genes are of betaproteobacterial origin. The genetic screening performed on samples from *P. peruvianus* and *P. madeirensis* allowed the amplification of five and four out of the six screened loci, respectively (Figure 1). This is consistent with previous descriptions of Phenacoccinae mealybug endosymbiotic systems (Husnik et al., 2013; Koga et al., 2013), suggesting that “*Ca*. Tremblaya phenacola” is the only endosymbiont in these species too. Unexpectedly, and in contrast with its recently described homolog in “*Ca*. Tremblaya phenacola” PAVE (Husnik et al., 2013), the *trpB* homologs identified in both *Phenacoccus* samples have best similarity hits with gammaproteobacterial proteins. Both sequences are highly similar but not identical (p-distance = 0.117), as expected for very closely-related orthologs. Additionally, the analysis of their nucleotide composition showed an AT-accumulation at codon degenerated positions (AT_N3_ = 80.2%, contrasting with AT_N1_ = 54.4% and AT_N2_ = 58%), which is common among obligate endosymbionts. Both facts appear to discard possible DNA contamination. To confirm the gammaproteobacterial origin of such sequences we performed a phylogenetic analysis using 771 unambiguously aligned sites of the *trpB* gene from 33 different prokaryote lineages including free-living and endosymbiotic bacteria from classes Beta and Gammaproteobacteria (Figure 2). Due to the short length of the aligned sequences, the topology, in some cases, does not reproduce the natural clades. Nevertheless, the sequences obtained from *P. peruvianus* and *P. madeirensis* are clearly located in the gammaproteobacterial clade.

### Determination of the location of the *trpB* gene in *Phenacoccus*

**Figure 3:**
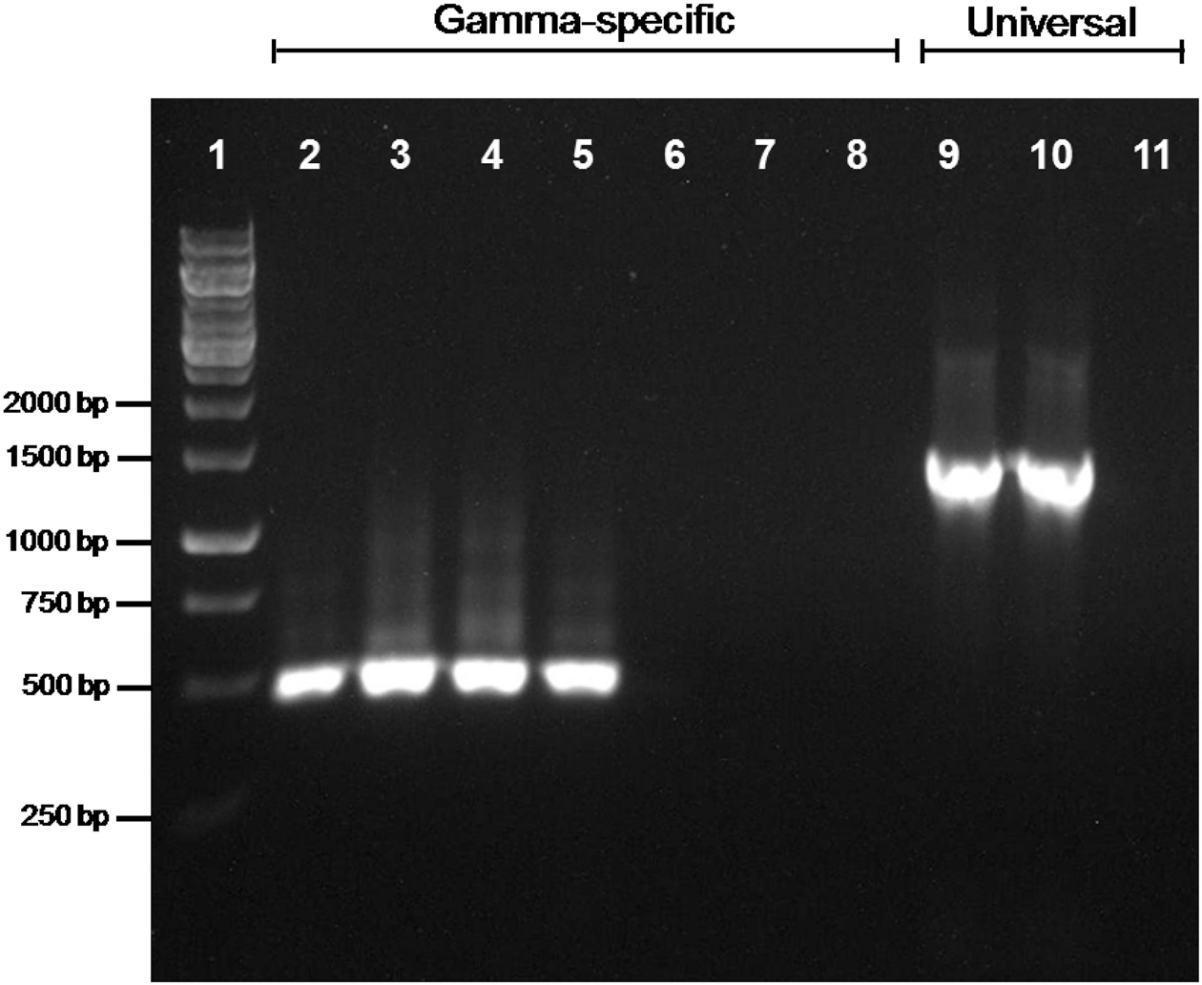
**PCR screening for gammaproteobacterial endosymbionts**. Gammaproteobacterial-specific primers were used on _T_DNA from *P. citri* (lane 2), *D. boninsis* (lane 3), *P. viburni* (lane 4), *P. longispinus* (lane 5), *P. peruvianus* (lane 6), and *P. madeirensis* (lane 7). The quality of samples from *P. peruvianus* and *P. madeirensis* was tested using 16S rDNA universal primers (lanes 9 and 10, respectively). Lanes 8 and 11 are the results of negative controls for each pair of primers.

**Figure 4:**
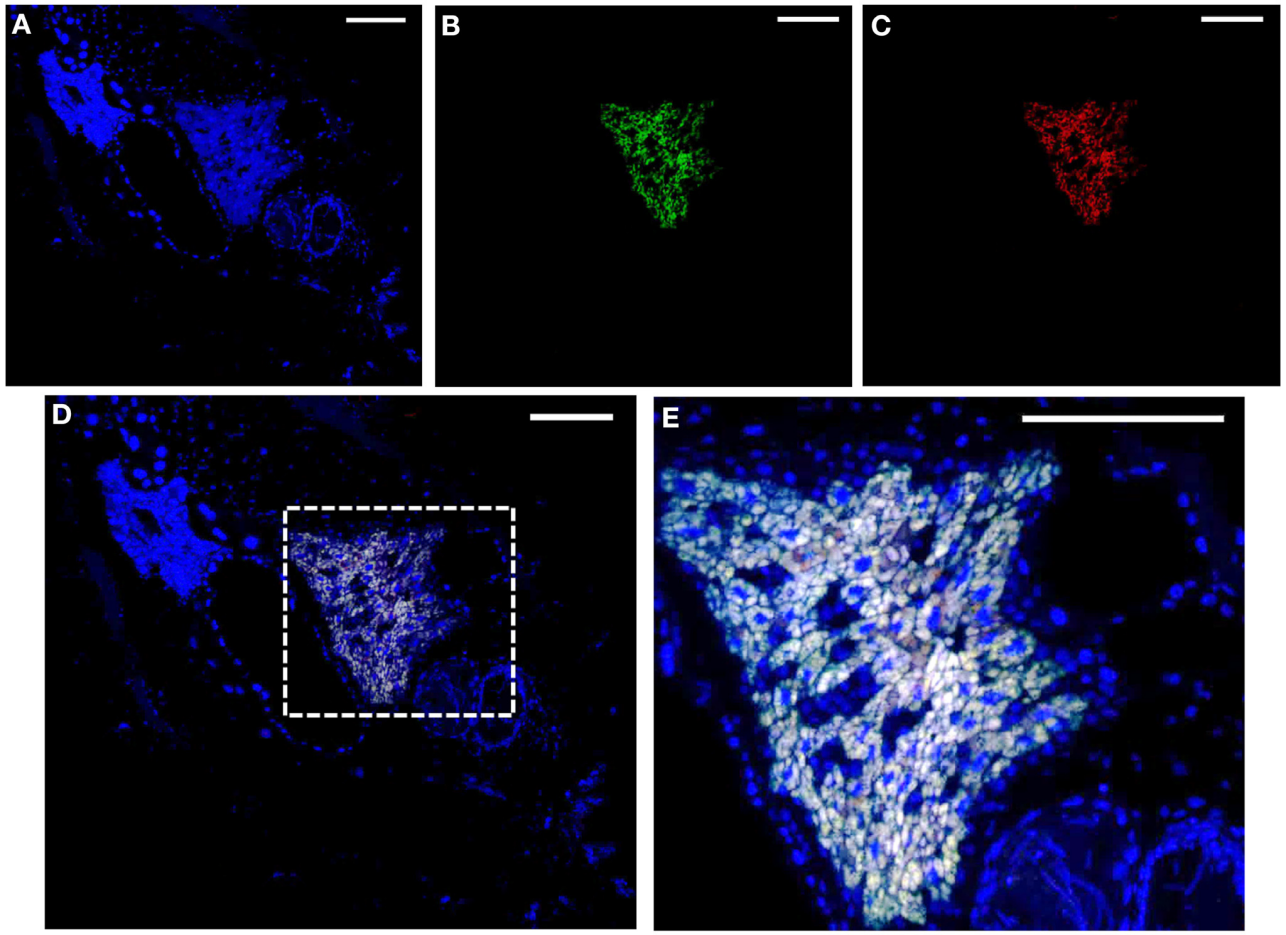
**FISH analysis of *P. peruvianus* bacteriome**. Adult female mealybugs stained with DAPI (blue: **A,D,E**) and probed with Cy5-EUB338 (green: **B,D,E**) and Cy3-TphPPER1290 (red: **C–E**). **(A–D)** Complete insect section showing a compact bacteriome. **(E)** Amplification of the region indicated in the dashed square in **(D)** to show the endosymbiotic system in more detail. Scale: 100 μm.

The amplification of a *trpB* gene of gammaproteobacterial origin might indicate that a second symbiont is also present in the two *Phenacoccus* species under study. To address this issue, we performed a 16S rRNA gene amplification on *P. peruvianus* and *P. madeirensis*. The PCR products were cloned, and 25 clones were sequenced, yielding a single sequence of betaproteobacterial origin from each mealybug species (1477 and 1467 bp, respectively). In order to search for the putative presence of low-density gammaproteobacteria in the tested samples, we performed a second PCR screening with gamma-specific primers. _T_DNA from the Pseudococcinae species *P. citri*, *D. boninsis*, *P. viburni*, and *P. longispinus*, where gammaproteobacterial endosymbionts had been detected, were used as positive controls. No gammaproteobacteria could be detected on the two *Phenacoccus* samples (Figure 3). The absence of a second bacterium was further confirmed by FISH analysis of *P. peruvianus* adult females. As it can be seen on Figure 4, the bacteriome appears as a well-defined organ inside the insect body cavity. It is visible under DAPI staining because DNA is found both in the nucleus (insect genome) and the cytoplasm (bacterial genomes). The only bacteria detected are exclusively located in the bacteriome, and the same fluorescent pattern is observed using both a universal probe and a “*Ca*. Tremblaya phenacola” specific probe.

**Figure 5:**
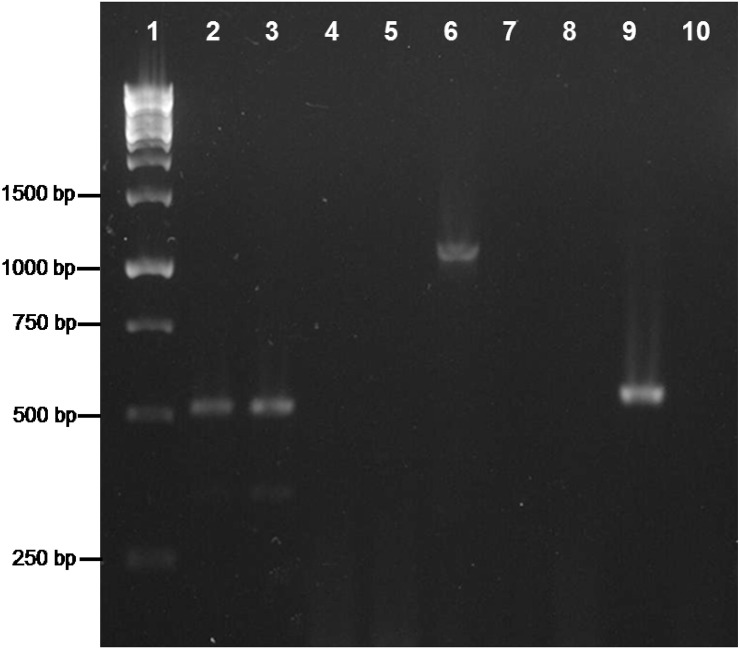
**PCR analysis on _T_DNA extracted from *P. peruvianus***. Heads (lines 2, 5, 8) and bodies (lines 3, 6, 9) were analyzed. Lane 1, Molecular Weight Marker; lines 2–4, amplification of 18S rDNA; lanes 5–7, amplification of the 16S rDNA; lanes 8–10, amplification of *trpB*. Negative controls: lanes 4, 7, 10.

To ascertain the location of the amplified *trpB* gene, either in the nuclear or the bacterial genome, we also followed a PCR approach (see Materials and Methods; Figure 5). The 18S rRNA gene, used as a positive control, was amplified in both head and body samples, while no amplification of the 16S rRNA gene was obtained from the head sample, thus confirming that it was not contaminated with bacteriocytes. As for the 16S rRNA gene, *trpB* was only amplified in the body samples, an indication that it is not present in the nuclear genome. Since no other bacterium was detected, *trpB* is likely to be located in the “*Ca*. Tremblaya phenacola” PPER genome.

### Molecular characterization and phylogenetic analysis of the endosymbionts from the analyzed mealybugs

**Figure 6:**
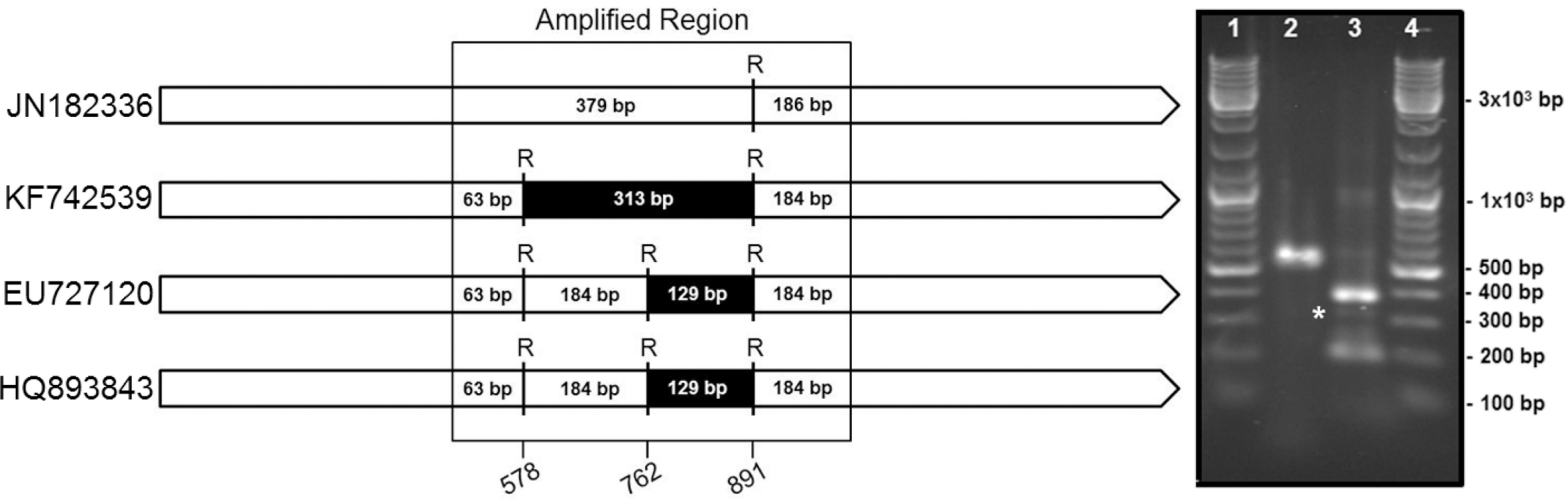
**Endosymbionts survey in *P. longispinus*. Left panel**: Restriction maps for the four putatively present 16S rDNA sequences, identified by their accession numbers in GenBank, i.e., “*Ca*. Tremblaya princeps” (JN182336) and the sequences of gamma-proteobacterial origin identified by us (KF742539), Duron et al. (2008) (EU727120), and Rosenblueth et al. (2012) (HQ893843). *Rsa*I restriction sites and expected fragments length are shown. Position numbers refer to the *E. coli* homolog. **Right panel**: RFLP results. Lanes 1 and 4, Molecular Weight Marker; lane 2, undigested amplicon; line 3, amplicon digested with *Rsa*I. A band around 313 bp (labeled with an asterisk) is indicative of the presence of the KF742539 sequence (this work). Absence of a band around 129 bp rules out the presence of EU727120 and HQ893843.

_T_DNA from *D. boninsis*, *P. longispinus*, *P. peruvianus*, and *P. madeirensis* was used for the amplification of 16S rDNA with universal primers, and the corresponding amplicons were cloned and sequenced. BLAST analysis of the obtained sequences revealed the presence of two different haplotypes, corresponding to a beta and a gammaproteobacterium, in the two Pseudoccocinae species (*D. boninsis* and *P. longispinus*), whereas the Phenacoccinae (*P. peruvianus* and *P. madeirensis*) yielded a single haplotype from a betaproteobacterium. We have obtained the almost-complete sequence of the 16S rRNA gene (1467 bp) of the betaproteobacterium from *P. madeirensis*, less than 50% of which was previously available (Gruwell et al., 2010). The sequence of betaproteobacterial origin amplified from *P. longispinus* is identical to the one that had been previously identified (Acc. no. JN182336). However, the gammaproteobacterial sequence obtained in this study (deposited in GenBank under Acc. no. KF742536) is not the same as the partial 16S rDNA sequences previously reported by Duron et al. (2008) and Rosenblueth et al. (2012). In fact, there are 13 polymorphic sites among the three haplotypes in the compared region (525 bp). A diagnostic screening by restriction enzyme analysis performed on the common region of the16S rRNA genes among “*Ca*. Tremblaya princeps” and the three gamma-haplotypes from *P. longispinus* confirmed that the sequences previously published were not present in our sample (Figure 6).

**Figure 7:**
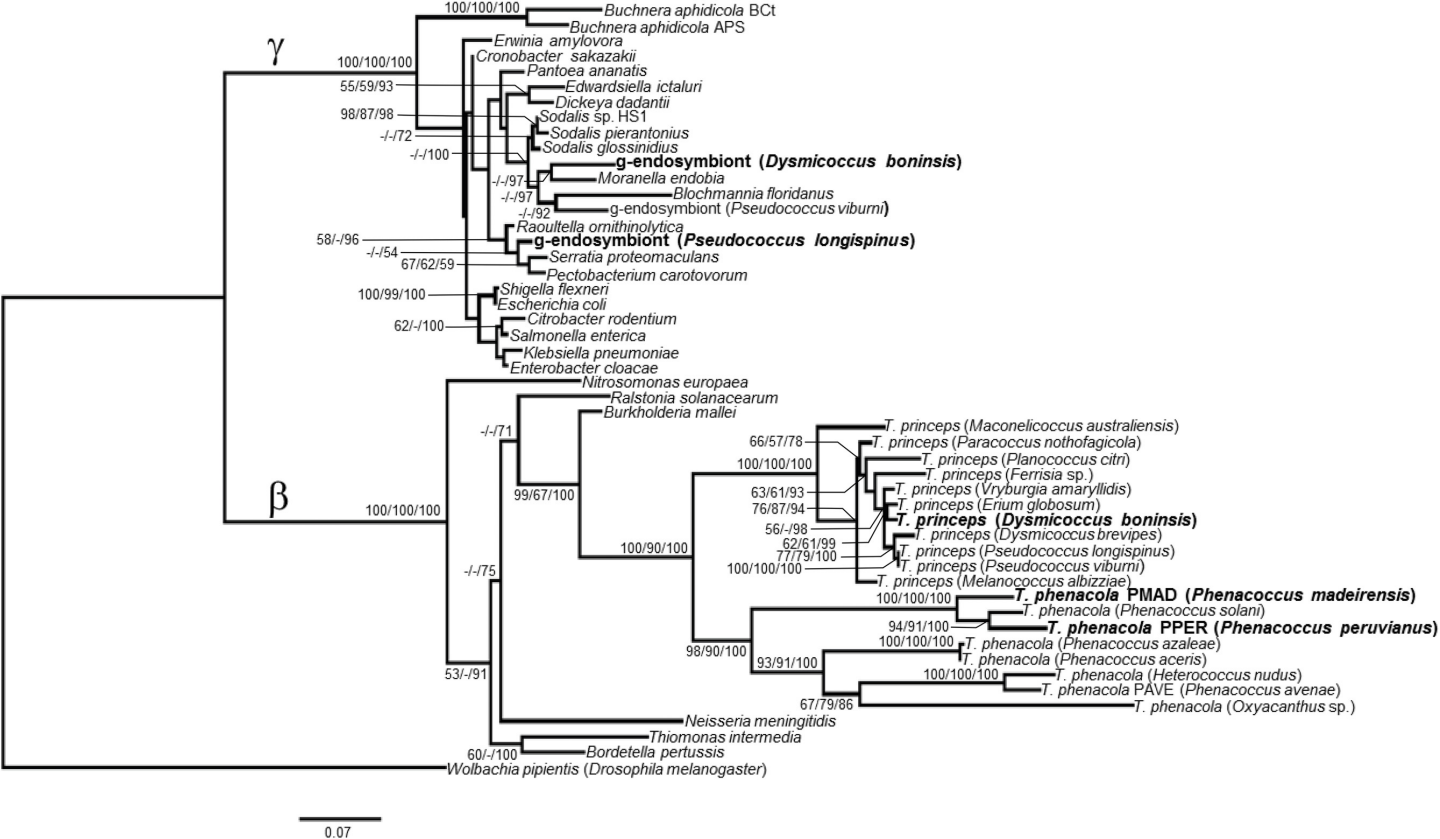
**ML phylogenetic analysis of gamma-endosymbionts and *Tremblaya* lineages based on their 16S rDNA sequences**. *Wolbachia pipientis* Dmel (alphaproteobacterial endosymbiont of *Drosophila melanogaster*) was used as outgroup. Bayesian and MP analysis gave essentially the same results. ML and MP bootstrap values, and Bayesian posterior probabilities over 50% are represented. Scale bar represents substitutions per site.

**Figure 8:**
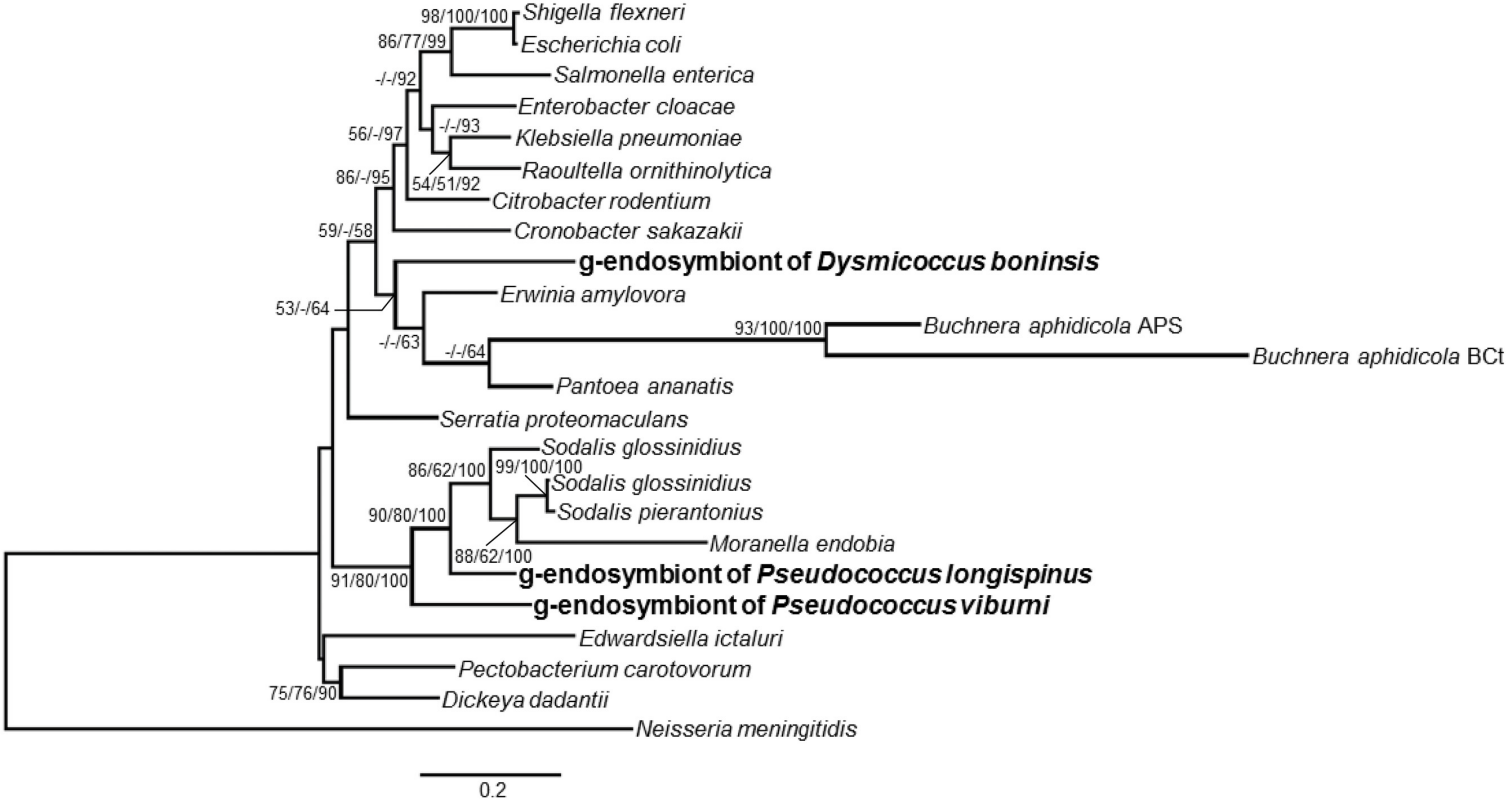
**Phylogenetic relationships among gamma-endosimbionts of mealybugs from subfamily *Pseudoccinae*, based on concatenated sequences of genes *argH* and *trpB***. Sequences obtained in this work are in bold. The betaproteobacterium *Neisseria meningitidis* MC58 was used as outgroup. Bayesian and MP analysis gave essentially the same results. ML and MP bootstrap values, and Bayesian posterior probabilities over 50% are represented. Scale bar represents substitutions per site.

The phylogenetic relationship of the endosymbionts under study was determined. The analysis was performed on 1221 unambiguously aligned sites of the 16S rRNA gene from 50 different prokaryote lineages, including gamma and betaproteobacteria, both free-living and endosymbionts (Figure 7). Two separate clades for beta and gammaproteobacteria are well defined. Among the beta-endosymbionts, the one from *D. boninsis* forms a monophyletic clade with the other “*Ca*. Tremblaya princeps,” while the endosymbionts of both *Phenacoccus* belong to the “*Ca*. Tremblaya phenacola” clade. They form a monophyletic cluster with the endosymbiont from *Phenacoccus solani* (Ferris), and appear separated from the subclade of strain PAVE. The obtained tree topology is congruent with that of the hosts (Hardy et al., 2008). Regarding the new gamma-endosymbionts characterized in this work, those from *D. boninsis* and *P. longispinus* do not cluster together. Nevertheless, these clustering have little support. To better define the phylogenetic position of the newly described gamma-endosymbionts, we took advantage of the *arg*H and *trp*B genes of gammaproteobacterial origin that we detected in the three Pseudococcinae mealybugs analyzed in this work (Figure 1). We performed a phylogenetic reconstruction using a concatenate of the available sequences from the two coding-genes for the same set of gamma-proteobacteria used in Figure 7. The analysis include 1308 unambiguously aligned sites (489 from *argH* and 819 from *trpB*) (Figure 8). This new tree confirms that the gamma-endosymbionts of *D. boninsis* and *P. longispinus* do not belong to the same clade. Furthermore, the gamma-endosymbionts of the two *Pseudococcus* species analyzed (*P. viburni* and *P. longispinus*) cluster with the *Sodalis*-like endosymbionts, but they do not form a monophyletic group either with “*Ca*. Moranella endobia” or between them.

Finally, we performed a molecular characterization of the 16S rDNA sequences from the analyzed beta-endosymbionts from the two *Phenacoccus* species under study. Strain PPER (from *P. peruvianus*) and PMAD (from *P. madeirensis*) have a GC-content of 48.5 and 48.1%, respectively. This GC-content fits well into the range described for the other characterized “*Ca*. Tremblaya phenacola” strains (from 45.8% in *Peliococcus turanicus* Kiritshenko to 50.6% in *Mirococcus sp*.), and it is clearly lower than the GC-content of the 16S rRNA gene from “*Ca*. Tremblaya princeps” (whose known minimum is 55.4% in *Pseudococcus comstocki* Kuwana; Koga et al., 2013). Multiple sequence alignment of the 16S rDNA sequences from “*Ca*. Tremblaya phenacola” strains PPER and PMAD revealed that both strains present four out of five motifs used by Gruwell et al. (2010) to define this species. However, in both cases the motif AGTT is modified to AGCT (positions 1240–1243 in the sequence from strain PPER). Additionally, the motifs AATGTC and TTTTA (sites 160–165 and 1121–1125, respectively, in PPER), also present in “*Ca*. Tremblaya phenacola” from *P. solani*, appear to be unique for this subclade members.

## Discussion

The availability of the complete “*Ca*. Tremblaya” genomes from two mealybug species revealed that “*Ca*. Tremblaya phenacola” PAVE alone is able to provide its host with the same essential amino acid biosynthetic capabilities as the consortium composed by “*Ca*. Tremblaya princeps” and “*Ca*. Moranella endobia” in *P. citri* (Husnik et al., 2013). However, no other mealybug species have been thoroughly analyzed for their essential amino acids biosynthetic capabilities. To address this issue, we performed a genetic screening in five endosymbiotic systems from different subclades of the mealybug subfamilies Pseudococcinae and Phenacoccinae. We screened for selected genes involved in the last step usually performed by the endosymbionts in the biosynthetic pathways of arginine (*argH*), branched amino acids (*ilvD* and *leuB*), methionine (*metE*), threonine (*thrC*), and tryptophan (*trpB*) (Figure 1). Many targeted genes were detected, which is consistent with the critical relevance of these bacteria in essential amino acid supply. Even though we were unable to obtain some amplicons, primer-pair amplification problems seem an improbable cause, because different beta and gammaproteobacteria were successfully amplified in the lineages under study, and no single DNA template or primer pair led to complete negative results. In any case, apparent absence of some genes in certain lineages should be interpreted carefully. Negative results do not necessarily imply the absence of a certain locus in the endosymbiotic system, although they might indicate the absence of a functional one. Since the degenerate primers were designed on gene regions encoding highly conserved residues, changes affecting the primer target sequences could affect both gene functionality and PCR results, potentially preventing the detection of pseudogenes. Nevertheless, we were able to amplify some likely recent pseudogenes that maintain a high level of identity with predicted functional homologs in closely related species and still retain most known critical residues for protein functioning (i.e., *argH* and *metE*). Functional redundancy tends to be lost following a stochastic process in endosymbiotic consortia, since only one copy is necessary to fulfill host needs. Therefore, the detected recent gene inactivation's, as well as the great variety in functional redundancies and gene retention patterns among endosymbionts of the Pseudococcinae lineages, suggest an ongoing specification of the role of each endosymbiotic partner in metabolic complementation. Moreover, our phylogenetic analyses (Figures 7, 8) indicate that each lineage was infected with different gammaproteobacteria, so that the detected gene losses are independent events. Our results indicate that all screened genes must have been present in the “*Ca*. Tremblaya” ancestor. Loss of *argH* apparently occurred after acquisition of the gammaproteobacterial partner in Pseudococcinae. At present, all analyzed species have retained an ortholog of gammaproteobacterial origin, but this gene appears to be functional in “*Ca*. Tremblaya princeps” from *D. brevipes* (Baumann et al., 2002), and it is pseudogenized in “*Ca*. Tremblaya princeps” from *P. citri*. *ilvD* has been retained by all “*Ca*. Tremblaya phenacola” analyzed, while Pseudococcinae shows various alternatives: in *P. citri* and *P. viburni*, it is only present in “*Ca*. Tremblaya princeps,” while in *D. boninsis* the gamma-endosymbiont performs this function. *leuB* has been retained in all “*Ca*. Tremblaya,” whereas it is redundant in *D. boninsis* and *P. longispinus*. *thrC* of gamma-endosymbiont origin was not detected in any analyzed lineage. *metE* is redundant in *D. boninsis* and *P. longispinus*, although it is pseudogenized in the gammaproteobacterium of the latter. The strict conservation of MetE key residues suggests that “*Ca*. Tremblaya princeps” performs the last step in methionine biosynthesis from cysteine in all surveyed Pseudococcinae, as well as in “*Ca*. Tremblaya phenacola” PAVE (Husnik et al., 2013). However, this gene was not detected in the two Phenococcinae analyzed here. The methionine synthase MetH (EC 2.1.1.13) may perform this last step in these endosymbionts, as in “*Candidatus* Hodgkinia cicadicola,” endosymbiont of cicadas (McCutcheon and Moran, 2010).

The most intriguing case relates to *trpB*. Even though Phenacoccinae harbor “*Ca*. Tremblaya phenacola” as a single endosymbiont, and in contrast to findings of the genome project of strain PAVE (Husnik et al., 2013), both *Phenacoccus* species analyzed here present only a gammaproteobacterial homolog (Figure 2). The two sequences we obtained are highly similar but not identical, and they present the common AT-content bias of P-endosymbionts. Both facts appear to discard DNA contamination. However, a PCR screening for gammaproteobacterial endosymbionts gave negative results for both *Phenacoccus* species (Figure 3). FISH and PCR analyses also showed that “*Ca*. Tremblaya phenacola” is the only bacteria found in *P. peruvianus*, where it is confined in the bacteriome (Figures 4, 5). Husnik et al. (2013) had recently reported several horizontally transferred genes of bacterial origin in the nuclear genome of *P. citri*, some of which are involved in the biosynthesis of several nutrients including the amino acid lysine. The source of such genes was not any of the members of the mealybug endosymbiotic consortium (i.e., “*Ca*. Tremblaya princeps” and “*Ca*. Moranella endobia”), even though many of them seem to complement gene loses in the consortium genomes. The authors suggest that several facultative symbionts, which are not essential for host survival, and can be free in the environment and infect the host sporadically, have been involved in HGT to the insect genome. Altogether, our findings suggest that HGT events have also occurred in mealybugs of the subfamily Phenacoccinae, affecting the evolution of, at least, one of the metabolic pathways for essential amino acids biosynthesis. In this case, however, the *trpB* gene appears to have been transferred to the “*Ca*. Tremblaya phenacola” genome. If confirmed by sequencing the whole endosymbiont genome, this would be (to our knowledge) the second case of HGT described in endosymbiotic bacteria. The other described case corresponds to “*Candidatus* Profftella armature,” a defensive symbiont from the psyllid *Diaphorina citri*, in which the genes involved in the biosynthesis of a cytotoxic metabolite appear to have been horizontally acquired (Nakabachi et al., 2013).

The analysis of the obtained 16S rDNA sequences (Figure 7) showed the presence of a beta-endosymbiont in all mealybug species under study, whereas in the Pseudococcinae there is also a gammaproteobacterium, as expected (Thao et al., 2002; Hardy et al., 2008; Gruwell et al., 2010). The phylogenetic reconstruction using a concatenate of *argH* and *trpB* allowed us a better characterization of the position of the newly identified gamma-endosymbionts (Figure 8). Our analyses show that both *Pseudococcus* gamma-endosymbionts analyzed in this work belong to the *Sodalis-like* clade. However, they do not form a monophyletic group either with “*Ca*. Moranella endobia” or between them. On the other hand, the gamma-endosymbiont of *D. boninsis* is not a *Sodalis*-like bacterium, consistently with the gamma-endosymbionts described for other species of the genus *Dysmicoccus*, based on 16S rDNA sequences (Thao et al., 2002).

The identification of the gamma-endosymbiont of *P. longispinus* had been controversial. Some authors were unable to detect it (Thao et al., 2002; Gatehouse et al., 2012), while two different haplotypes have been identified in other studies (Duron et al., 2008; Rosenblueth et al., 2012). In all these cases the genetic screening was not exhaustive (due to the analysis of a limited number of clones), and the amplified sequences were shorter than the one obtained in this work. Our results, based on the analysis of 36 clones, indicate the presence of a single gamma-endosymbiont in this species, which is consistent with what had been found in other Pseudococcinae. However, our sequence does not correspond to any of the previously described (Figure 6). The existence of different haplotypes might indicate high levels of intraspecific polymorphisms in the gamma-endosymbionts of *P. longispinus*. Alternatively, due to the high morphological similarity among mealybug species, problems in the identification of insect host species cannot be ruled out.

We have also performed phylogenetic and molecular characterization of “*Ca*. Tremblaya phenacola” strains PPER and PMAD. Only a few strains have been reported in this species previously, and two subclades have been described (Gruwell et al., 2010). As revealed by the phylogenetic analysis based on 16S rDNA sequences (Figure 7, Table S3 in Supplementary Material), strains PPER and PMAD are members of the most unexplored subclade. While they present most of the characteristic sequences used by Gruwell et al. (2010) to define this species, we also identified some unique molecular signatures for this subclade. Considering the high morphological similarity exhibited by mealybug species, these sequences could be useful as potential targets on strategies for both bacteria and insect molecular identification (Cox, 1983; Charles et al., 2000). Specifically, the mealybug species *P. solani*, *P. peruvianus*, and *P. madeirensis*, which belong to this subclade and are invasive pests of horticultural and ornamental plants, represent a relevant threat in several European countries (Pellizzari and Germain, 2010). Therefore, the ability to differentiate them, at the molecular level, from other widespread polyphagous species such as *P. citri*, *P. viburni*, and *P. longispinus*, could provide a rapid pests detection tool for import/export controls in Europe.

In summary, our molecular and phylogenetic analyses provide a more complete picture of the complex evolutionary history of the two “Ca. Tremblaya” lineages. The genetic screening of selected genes confirmed the importance of mealybug endosymbionts in providing essential amino acids to their hosts. In several Pseudococcinae analyzed, the complementation of “*Ca*. Tremblaya princeps” and the gamma-endosymbionts is ongoing, given the gene redundancies found. We have also identified a putative case of HGT in “*Ca*. Tremblaya phenacola” for the biosynthesis of tryptophan. Finally, from an applied point of view, two diagnostic motifs in the 16S rDNA sequence have been identified, which could be potentially used to implement a rapid detection method to differentiate mealybug pests in horticultural and ornamental plants.

### Conflict of interest statement

The authors declare that the research was conducted in the absence of any commercial or financial relationships that could be construed as a potential conflict of interest.
