# Supplementary material for: Molecular evidence for ongoing complementarity and horizontal gene transfer in endosymbiotic systems of mealybugs
Source: Front Microbiol. 2014 Aug 26;5:449. doi: 10.3389/fmicb.2014.00449 (PMC4144094; doi:10.3389/fmicb.2014.00449)
Supplement: Supplementary file 1 [file DataSheet1.DOCX]

**SUPPLEMENTARY MATERIALS**

**Table S1. Bacterial species included in the multiple alignment leading to degenerate primers design**

| **Bacterial Species** | **Class*** | **Accession Numbers** |
| --- | --- | --- |
| *Burkholderia glumae* BGR1 | Beta | CP001503, CP001504 |
| *Burkholderia multivorans* ATCC 17616 | Beta | AP009385, AP009386, AP009387 |
| *Burkholderia pseudomallei* 1106a | Beta | CP000572, CP000573 |
| *Burkholderia thailandensis* E264 | Beta | CP000085, CP000086 |
| *“Candidatus* Tremblaya princeps” PCVAL | Beta | CP002918 |
| *Escherichia coli* K-12 substr. MG1655 | Gamma | U00096 |
| *Salmonella enterica* Typhimurium str. LT2 | Gamma | AE006468 |
| *Serratia proteomaculans* 568 | Gamma | CP000826 |
| *Sodalis glossinidius* | Gamma | AP008232 |
| *Dickeya dadantii* 3937 | Gamma | CP002038 |
| *Yersinia pestis* Angola | Gamma | CP000901 |
| *“Candidatus* Moranella endobia” PCVAL | Gamma | CP003881 |

*Beta: betaproteobacteria; Gamma: gammaproteobacteria

**Table S2.** List of additional primers used in this work

| **Gene** | **Primer pairs** | **Sequence (5’→3’)** | **Specificity** | **Insect sample** | **Application**  **(Results section)** | **Reference** |
| --- | --- | --- | --- | --- | --- | --- |
| 16S | 16S-up  16S-down | AGAGTTTGATCATGGCTCAGATTG  TACCTTGTTACGACTTCACCCCAG | Bacteria | *D. boninsis*  *P. longispinus*  *P. peruvianus*  *P. madeirensis* | Endosymbionts molecular characterization  (3.3) | van Ham *et al*., 1997 |
| 16S | Gamma395f  Gamma871r | CMATGCCGCGTGTGTGAA  ACTCCCCAGGCGGTCDACTTA | γ-proteobacteria | *P. peruvianus*  *P. madeirensis* | Endosymbionts molecular characterization  (3.2) | Mühling *et al*., 2008 |
| 18S | 930F  1270R | GCATGGAATAATGGAATAGG  CCGTCAATTCCTTTAAGT | Eukarya | *P. peruvianus* | trpB localization (3.2) | Littlewood and Olson, 2001 |
| 16S | HGT-16-F  HGT-16-R | GGTGGCTAATACCGCATAATGTC  GGCTAGTTCTTACGAAGTTGCAG | “*Ca*. Tremblaya phenacola” from *P. peruvianus* | *P. peruvianus* | trpB localization (3.2) | This work |
| *trpB* | HGT-TRPB-F  HGT-TRPB-R | CATGGTGTGGCTTCTTCAATAG  GGAAGTCAAGACCAGCAGATA | “*Ca*. Tremblaya phenacola” from *P. peruvianus* | *P. peruvianus* | trpB localization (3.2) | This work |
| 16S | RFLP-up  RFLP-down | TGCCAGCAGCCGCGGTAATAC  ACACGAGCTGACGACAGCCATG | Endosymbionts of *P. longispinus* | *P. longispinus* | RFLP analysis  (3.3) | This work |
|  |  |  |  |  |  |  |

**Table S3. Bacterial species included in the phylogenetic analyses, by alphabetical order in each class.** Accession numbers refer to complete genomes or available sequences for 16S rRNA^1^, *argH^2^* and *trpB^3^* genes. Sequences obtained in this work appear in bold.

| **Species** | **Class** | **Insect host** | **Accession number** |
| --- | --- | --- | --- |
| *Wolbachia* *pipientis* | Alpha | *Drosophila melanogaster* | AE017196 |
| *Bordetella pertussis* CS | Beta | - | CP002695 |
| *Burkholderia mallei* ATCC 23344 | Beta | - | CP000011 |
| *“Ca.* Tremblaya phenacola*”* | Beta | *Phenacoccus solani* | HM449980^1^ |
| *“Ca.* Tremblaya phenacola*”* | Beta | *Phenacoccus aceris* | HM449982^1^ |
| *“Ca.* Tremblaya phenacola*”* | Beta | *Phenacoccus azaleae* | AB627026^1^ |
| *“Ca.* Tremblaya phenacola*”* | Beta | *Heterococcus nudus* | HM449976^1^ |
| *“Ca.* Tremblaya phenacola*”* | Beta | *Oxyacanthus sp.* | HM449972^1^ |
| *“Ca.* Tremblaya phenacola*”* PAVE | Beta | *Phenacoccus avenae* | CP003982 |
| *“Ca.* Tremblaya phenacola*”* PMAD | Beta | *Phenacoccus madeirensis* | **KF444180**^1^, **KF444184**^3^ |
| *“Ca.* Tremblaya phenacola*”* PPER | Beta | *Phenacoccus peruvianus* | **KF444174**^1^, **KF444179**^3^ |
| *“Ca.* Tremblaya princeps*”* | Beta | *Maconellicoccus australiensis* | AF476088^1^ |
| *“Ca.* Tremblaya princeps*”* | Beta | *Melanococcus albizziae* | AF476087^1^ |
| *“Ca.* Tremblaya princeps*”* | Beta | *Paracoccus nothofagicola* | AF476094^1^ |
| *“Ca.* Tremblaya princeps*”* PCVAL | Beta | *Planococcus citri* | CP002918 |
| *“Ca.* Tremblaya princeps*”* | Beta | *Ferrisia* sp. | AF476086^1^ |
| *“Ca.* Tremblaya princeps*”* | Beta | *Vryburgia amaryllidis* | AF476097^1^ |
| *“Ca.* Tremblaya princeps*”* | Beta | *Erium globosum* | AF476084^1^ |
| *“Ca.* Tremblaya princeps*”* | Beta | *Pseudococcus longispinus* | AF476093^1^ |
| *“Ca.* Tremblaya princeps*”* | Beta | *Dysmicoccus brevipes* | AF476082^1^ |
| *“Ca.* Tremblaya princeps*”* | Beta | *Pseudococcus viburni* | JN182337^1^ |
| *“Ca.* Tremblaya princeps*”* | Beta | *Dysmicoccus boninsis* | **KF911099**^1^ |
| *Neisseria meningitidis* MC58 | Beta | - | AE002098 |
| *Nitrosomonas europaea* ATCC 19718 | Beta | - | AL954747 |
| *Ralstonia solanacearum* GMI1000 | Beta | - | NC003295 |
| *Thiomonas intermedia* K-12 | Beta | - | CP002021 |
| *Blochmannia floridanus* | Gamma | *Camponotus floridanus* | NC_005061 |
| *Buchnera aphidicola* APS | Gamma | *Acyrthosiphon pisum* | NC_002528 |
| *Buchnera aphidicola* BCt | Gamma | *Cinara tujafilina* | NC_015662 |
| *“Ca.* Moranella endobia*”* PCVAL | Gamma | *Planococcus citri* | CP003881 |
| *Citrobacter rodentium* ICC168 | Gamma | - | FN543502 |
| *Cronobacter sakazakii* ATCC BAA-894 | Gamma | - | NC_009778 |
| *Dickeya dadantii* 3937 | Gamma | - | NC_014500 |
| *Edwardsiella ictaluri* 93-146 | Gamma | - | NC_012779 |
| *Enterobacter cloacae* ATCC 13047 | Gamma | - | NC_014121 |
| *Erwinia amylovora* ATCC 49946 | Gamma | - | FN666575 |
| *Escherichia coli* K-12 MG1655 | Gamma | - | U00096 |
| *Klebsiella pneumoniae* 342 | Gamma | - | CP000964 |
| *Pantoea ananatis* AJ13355 | Gamma | - | NC_017531 |
| *Pectobacterium carotovorum* subsp. carotovorum PC1 | Gamma | - | NC_012917 |
| *Raoultella ornithinolytica* B6 | Gamma | - | NC_021066 |
| *Salmonella enterica* DT104 | Gamma | - | HF937208 |
| *Serratia proteamaculans* 568 | Gamma | - | CP000826 |
| *Shigella flexneri* 2a str. 301 | Gamma | - | NC_004337 |
| *Sodalis* sp. HS1 | Gamma | - | CP006569 |
| *Sodalis glossinidius* | Gamma | *Glossina morsitans* | AP008232 |
| *Sodalis pierantonius* SOPE | Gamma | *Sitophilus oryzae* | CP006568 |
| Unnamed endosymbiont | Gamma | *Dysmicoccus boninsis* | **KF911098**^1^, **KF444196**^2^, **KF444200**^3^ |
| Unnamed endosymbiont | Gamma | *Pseudococcus longispinus* | **KF742539**^1^, **KF444192**^2^, **KF444195**^3^ |
| Unnamed endosymbiont | Gamma | *Pseudococcus viburni* | JN182341^1^**,**  **KF444190**^2^, **KF444191**^3^ |

**SUPPLEMENTARY RESULTS**

***Analysis of pseudogenes involved in the biosynthesis of essential amino acids.*** The multiple alignment of homolog loci from a set of selected betaproteobacteria (*Burkholderia mallei* NCTC10229, *“Ca. Tremblaya princeps”* from *D. brevipes*, *Ralstonia solanacearum* GMI1000, *Nitrosomonas europea*, *Neisseria meningitidis* MC58, *Thiomonas intermedia* K-12 and *Bordetella pertusis*), and *E. coli* allowed us to identify the inactivating mutations in the *argH* locus in “*Ca*. Tremblaya princeps” PCVAL (GenBank acc. no. CP002918) and the *metE* locus in the gamma-endosymbiont from *P. longispinus* (GenBank acc. no. KF444194).

*argH*. We have identified two deletions, involving 6 and 57 nucleotides (sites 37548-49 and 37890-91, respectively), which cause the loss of the fully conserved amino acids A56, Y172, M175, R178, D179 and R182 (amino acid numbers refer to the homolog protein in *E. coli*). We also detected an inactivating frameshift caused by a single cytosine deletion between sites 38038 and 38039, which is part of the GCG codon (A236) in the homolog gene of *“Ca.* Tremblaya princeps*”* from *D. brevipes* (Baumann *et al*., 2002). However, two protein functional domains (PF00206 and PF14698 at sites 37402-38222 and 38409-38594, respectively) and their predicted active residues (H156, S277 and E290) are still recognizable in the *“Ca.* Tremblaya princeps*”* PCVAL pseudogene, according to Pfam information.

*metE*. The *metE* homolog in the *P. longispinus* gamma-endosymbiont appears to be inactivated due to the presence of a nonsense mutation (TGG→TAG) affecting the highly conserved W140 in the *E. coli* homolog protein. However, a careful analysis of all known residues that are important for protein function (Pejchal *et al*., 2005; Koutmos *et al*., 2008) showed that they are still preserved, as in all other mealybug endosymbionts analyzed, which indicates a recent pseudogenization event. The results of the essential motif analysis are presented in Table S4.

The methionine synthase MetE (EC 2.1.1.13) catalyzes the transfer of a methyl group from N5-methyl-5,6,7,8-tetrahydrofolate to L-homocysteine, activating L-homocysteine by binding the thiolate form of the substrate to Zn^2+^. The motifs analyzed in this work include the four residues at the zinc-binding site, the residues that participate at the zinc-replete MetE-L-homocysteine complex formation, those that participate at the generation of the MetE-N5-methyl-5,6,7,8-tetrahydrofolate complex, and the DMV region involved in the substrate binding sites communication, so that the binding of any of the substrates increases MetE affinity for the acquisition of the other substrate.

Table S4. Summary of residues involved in MetE enzymatic function

|  |  | *P. avenae* |  | *P. citri* |  | *D. boninsis* | |  | *P. viburni* |  | *P. longispinus* | |
| --- | --- | --- | --- | --- | --- | --- | --- | --- | --- | --- | --- | --- |
| MetE functional motifs | *E. coli* key amino acids | β |  | β |  | β | γ |  | β |  | β | γ |
| Zn2+ Site | H641 | + |  | + |  | + | + |  | + |  | + | + |
|  | C643 | + |  | + |  | + | + |  | + |  | + | + |
|  | E665 | + |  | + |  | + | + |  | + |  | + | + |
|  | C726 | + |  | + |  | n. a. | n. a. |  | n. a. |  | n. a. | n. a. |
| MetE-Hcy binary complex | I431 | V |  | V |  | V | + |  | V |  | V | + |
|  | S433 | + |  | + |  | + | + |  | + |  | + | + |
|  | E484 | + |  | + |  | + | + |  | + |  | + | + |
|  | M490 | + |  | + |  | + | + |  | + |  | + | + |
|  | D599 | + |  | + |  | + | + |  | + |  | + | + |
| MetE-folate binary complex | R17 | + |  | + |  | n. a. | n. a. |  | n. a. |  | n. a. | n. a. |
|  | K20 | + |  | + |  | n. a. | n. a. |  | n. a. |  | n. a. | n. a. |
|  | K117 | + |  | + |  | n. a. | n. a. |  | n. a. |  | n. a. | n. a. |
|  | R515 | + |  | + |  | + | + |  | + |  | + | + |
|  | K518 | + |  | + |  | + | + |  | + |  | + | + |
|  | W561 | + |  | + |  | + | + |  | + |  | + | + |
|  | E605 | + |  | + |  | + | + |  | + |  | + | + |
| DMV sequence | D489 | + |  | + |  | + | + |  | + |  | + | + |
|  | M490 | + |  | + |  | + | + |  | + |  | + | + |
|  | V491 | + |  | + |  | + | + |  | + |  | + | + |

Amino acid numbers refer to the *E. coli* K12 *substr.* MG1655 homolog. Since *metE* sequences are only partially available for *D. boninsis*, *P. viburni* and *P. longispinus* endosymbionts, information about the conservation of R17, K20, K117 and C726 homolog residues is not available (n. a.). Full identity was detected for all analyzed amino acids except for I431 (involved in MetE-Hcy binary complex formation), turned into the also non-polar amino acid valine in all analyzed *Tremblaya* lineages.

**REFERENCES**

Baumann, L., Thao, M.L., Hess, J.M., Johnson, M.W., and Baumann, P. (2002). The genetic properties of the primary endosymbionts of mealybugs differ from those of other endosymbionts of plant sap-sucking insects. *Appl. Environ. Microbiol*. 68, 3198–3205. doi: 10.1128/AEM.68.7.3198

Koutmos, M., Pejchal, R., Bomer, T.M., Matthews, R.G., Smith, J.L., and Ludwig, M.L. (2008). Metal active site elasticity linked to activation of homocysteine in methionine synthases. *Proc. Natl. Acad. Sci*. 105, 3286–3291. doi: 10.1073/pnas.0709960105

Pejchal, R., and Ludwig, M.L. (2005). Cobalamin-independent methionine synthase (MetE): a face-to-face double barrel that evolved by gene duplication. *PLoS Biol*. **3**:2. doi: 10.1371/journal.pbio.0030031
